# Patterns of globalized reproduction: Egg cells regulation in Israel and Austria

**DOI:** 10.1186/2045-4015-1-15

**Published:** 2012-04-18

**Authors:** Carmel Shalev, Gabriele Werner-Felmayer

**Affiliations:** 1 Department for Reproduction and Society, International Center for Health, Law and Ethics, Haifa University, Israel; 2 Division of Biological Chemistry, Biocenter, and Bioethics Network Ethucation, Medical University of Innsbruck, Fritz-Pregl-Strasse 3, A-6020 Innsbruck, Austria

## Abstract

Since the successful introduction of *in vitro *fertilization in 1978, medically assisted reproduction (MAR) has proliferated in multiple clinical innovations. Consequently, egg cells have become an object of demand for both infertility treatment and stem cell research, and this raises complex legal, ethical, social and economic issues.

In this paper we compare how the procurement and use of human egg cells is regulated in two countries: Israel and Austria. Israel is known for its scientific leadership, generous public funding, high utilization and liberal regulation of assisted reproductive technology (ART). Austria lies at the other extreme of the regulatory spectrum in terms of restrictions on reproductive interventions.

In both countries, however, there is a constant increase in the use of the technology, and recent legal developments make egg cells more accessible. Also, in both countries the scarcity of egg cells in concert with the rising demand for donations has led to the emergence of cross-border markets and global 'reproductive tourism' practices. In Israel, in particular, a scandal known as the 'eggs affair' was followed by regulation that allowed egg cell donations from outside the country under certain conditions.

Cross-border markets are developed by medical entrepreneurs, driven by global economic gaps, made possible by trans-national regulatory lacunae and find expression as consumer demand. The transnational practice of egg cell donations indicates the emergence of a global public health issue, but there is a general lack of medical and epidemiological data on its efficacy and safety. We conclude that there is need for harmonisation of domestic laws and formulation of new instruments for international governance.

## Introduction

Since the successful introduction of *in vitro *fertilization in 1978, medically assisted reproduction (MAR) has proliferated in multiple clinical innovations. Consequently, egg cells have become an object of demand for both infertility treatment and stem cell research. However, the procurement of egg cells involves conflicting interests and raises multiple concerns and ethical issues. In this paper we compare the approach to egg cell donations in two countries with populations of comparable size (around 8 million): Israel and Austria. First we provide a general background on the medical practice of egg cell donations for infertility therapy, the commercialization of reproductive medicine and the concerns about the commodification of women's body parts, given the intrinsic scarcity of egg cells and the growing demand for egg cells both for reproduction and for stem cell research. Then we compare ART utilization rates in Israel and Austria, and the different regulatory approaches. Finally we focus on the emergence of cross-border markets in reproductive tourism, in which egg cells become a coveted commodity, as a result of economic gaps and differences in law and policy between countries. We conclude that there is need for harmonisation of domestic laws and formulation of new instruments for international governance.

## Egg cells

### Third-party reproduction

Since the birth of the first *in vitro *fertilisation (IVF) baby, Louise Brown, in 1978, it is estimated that 3.75 million babies have been born world wide with the assistance of medical reproductive technologies [1]. In 2010 the Nobel Prize was awarded to the embryologist Robert Edwards for his pioneering work on IVF, noting that its development was "a medical advance that represents a paradigm shift in the treatment of many types of infertility" [2]. Indeed, IVF was introduced as a treatment for female infertility in the case of blocked fallopian tubes, but by the mid-1990s it had become standard treatment for male infertility together with intra-cytoplasmic sperm injection (ICSI)^1^, as well as for cases of infertility with unknown cause [1]. IVF is also utilised with third-party reproduction practices (sperm donors, surrogate mothers and egg cell donors), primarily to address medical indications of infertility in heterosexual couples, and subsequently to overcome non-medical obstacles to pregnancy and parenthood, for singles and same sex couples.

Like in the case of IVF, medical indications for egg cell^2 ^donation expanded rapidly. At first, it was indicated as treatment for female infertility due to ovulatory disorders. It soon came to be used also in cases of genetic and mitochondrial mutations. In the latter decade, assisted reproductive technology (ART) with egg cells donation has been utilised increasingly by menopausal women who can still carry pregnancies. Since egg quality is the primary barrier to pregnancy in older women [3], egg cell donations from younger women have come to be perceived as a means to extend the child-bearing years of older women in their late forties and fifties. In the course of these developments, a market has formed around the need for egg cells, and they have emerged as a coveted commodity.

Many of these developments have been driven by private medical entrepreneurs. A major factor in the rapid proliferation of assisted reproductive technology (ART) was its burgeoning in the USA in what has been described as a 'Wild West' of unregulated research, characterized by an almost instantaneous translation of experimental procedures into marketed services [4]. In recent years, the commercialization of reproductive medicine has become evident also in the emergence of transnational markets in reproductive tourism, in which women are both buyers and sellers, including for the purpose of egg cell 'donation'.

Who are the donors? Websites boast that they do not accept all candidates and pride themselves on their connection with university students, which implies that their potential donors are young, intelligent and of relatively high socio-economic status. Candidates are screened for medical counter-indications, such as reproductive disorders or genetic disease. They are described in terms of personality traits, talents and hobbies. Physical features are also marketed, either to match the looks of the prospective egg cell recipient, or a eugenic model of weight, height, hair, eye color and race, according to the preferences of the recipients [5]. The websites also recruit the donors. They persuade women by offering them significant sums of money, and appealing to their altruism and generosity to help another woman to get pregnant, experience childbirth and have a child, even if she cannot bear genetic offspring. At the end of the day, though, selling egg cells is not a respected occupation. Norms of anonymity transform the intimacy of reproductive relations into a cold business transaction, create secrecy, shame and taboo, and make it all too easy to objectify the egg donor - as if 'the lady vanishes' [6].

While there is a degree of market activity around sperm donation as well, major concerns arise in relation to the exploitation of women and the commodification of female body parts. Male and female reproductive factors are not analogous: sperm is abundant, easily obtainable and inexpensive; whereas egg cells are a scarce resource and their procurement entails risky and invasive intervention in the woman's body. In the context of third-party reproduction, the female body and its reproductive parts are of much higher market value than the male one. Interestingly enough, the need for eggs seems to be greater than for wombs, and the commerce in egg cells in recent years seems to have outgrown the surrogacy market. In Israel, for example, the number of requests to approve surrogacy agreements, over a period of fifteen years, is in the range of several hundreds.^3 ^On the other hand, during the parliamentary discussions of the Eggs Donation Law, 2010, estimates of the number of women seeking egg cell donations each year were in the thousands. Meanwhile, stem cell research for regenerative medicine presents an additional demand for egg cells that competes with the need for reproductive purposes [7].

### Scarcity and delicacy

Human egg cells were not known as such until their discovery by the embryologist Karl Ernst von Baer in 1826. At the time, they were thought to be a mere vessel for the male semen, and their essential contribution to embryo formation was not acknowledged until the second half of the 19^th ^century [8]. Today we know the details of the sophisticated biological processes regulated by hormones which lead to the formation of mature egg cells in women of reproductive age. It seems that the entire pool of immature egg cells is built up prenatally, peaking at the 20^th ^week of fetal development. Afterwards, a process termed atresia systematically reduces their amount from 6 to 7 million to about 2 million at birth, and even further to about 250,000 by the start of menstruation. During a woman's reproductive years, usually one more or less immature egg cell will embed in the follicle per cycle,^4 ^and only between 300 and 500 egg cells will mature overall to a stage in which they can be fertilized. This intricately regulated process starts to wind down around the age of 40 and finally stops around the age of 50. The ovarian ageing process affects both the quantity and quality of egg cells, and this in turn results in increased rates of chromosomal disorders making pregnancy impossible in the majority of cases or causing more or less severe birth defects. Numerous factors can disturb the complex processes of ovarian function which may lead to premature ovarian failure and infertility [9,10].

In other words, the mature egg cell is a precious natural specimen for each woman. Because in most natural cycles only a single follicle matures, it has become standard IVF procedure to administer hormonal treatment so as to induce artificial maturation of more than one egg cell, and to stimulate the release of a large number of eggs in any one menstrual cycle. However, the risks of intervention for egg cell procurement are substantial. First, the hormonal treatment regimens can carry adverse reactions. Ovarian hyper-stimulation syndrome, a potentially life-threatening condition, is one of the more prominent immediate risks, while knowledge of the long-term effects is limited so far. Milder stimulation protocols are less risky and show cumulative pregnancy rates that are comparable to standard protocols, but may require more cycles of treatment [11-13]. In addition, the egg retrieval procedure itself is intrusive and requires local or general anesthesia. (See Additional File 1 for a description of the health risks in Israel's standard IVF informed consent form.)

At the same time, commercialized medical practices are often geared towards maximizing productivity or optimizing yields, and are driven by 'competition for patients, desire for high fertility rates and the need for quick results', at the expense of donor health [13]. Because many of these practices take place in the private market, there is inadequate epidemiological data on the risks for egg donors, but anecdotal testimonies indicate that they can be substantial [14]. One UK press report brought the stories of two women who claimed to have suffered health damage after donating eggs at a clinic in Bucharest which was collaborating with a private clinic in London. One of the women, aged 18 at the time of the donation, was left with scarred ovaries that rendered her infertile [15].

### Stem cell research

Meanwhile, egg cells have also become a coveted commodity in embryonic stem cell research for the purpose of personalised regenerative medicine (i.e., the potential production of homologous tissue or organs for transplantation in sick persons). Any progress in this area will translate rapidly into economic profit long before it reaches the patient's bedside, and this creates a conflict of interest that can compromise research integrity.

This came to light in 2005, in what came to be known as the Korean stem cell scandal. The affair involved a veterinarian scientist who had published two papers in *Science *magazine in which he claimed to have successfully extracted stem cells from human embryos cloned by means of somatic cell nuclear transfer (SCNT).^5 ^Allegations of scientific misconduct were exposed by journalists and led to an independent investigation conducted by Seoul National University, which found that the scientist had intentionally fabricated research results. Consequently *Science *retracted the fraudulent publications. The investigation also found ethical misconduct in the appropriation of the human egg cells that were used for the cloning research. The scientist claimed to have used a new technique that reduced the need for eggs. However, contrary to his claim of having used 185 eggs, according to research records kept in his laboratory at least 273 eggs were shown to have been used [16]. Korea's National Bioethics Board found that he had used a total of 2,221 eggs from 119 women, and there were serious shortcomings in the process of obtaining informed consent from them [17]. Among other things, the research team did not properly inform women about the health risks involved in the follicle stimulation protocols and the egg cell procurement procedure, junior members of the research team were pressured into providing egg cells, and researchers had paid donors although the consent form stated that they had received no financial payment [7,18].

Following this scandal SCNT research fell into some disrepute, while scientists' interest shifted to a certain degree from embryonic to adult stem cells. Alternative methods have been established to achieve induced pluripotent cell lines^6 ^from adult skin cells, without using egg cells or embryos, and research in this field is progressing rapidly [19]. Nonetheless, the SCNT approach is still a subject of strong research interest [20]; and egg cells remain an important raw material for research in reproductive and regenerative medicine. There is also interest in parthenogenic blastocysts^7 ^for use in potential cell therapies. Patient-specific transplantation tissues and organs or 'personalised' stem cell therapies, with either SCNT embryos or parthenogenic blastocysts, would require large numbers of egg cells for translation into clinical practice. If such research proves to have clinical application, the availability of sufficient numbers of egg cells will become a major problem and is likely to exceed the demand generated by infertility treatments [21].

Another experimental technique that is intended to 'repair' egg cells of IVF patients illustrates both the reciprocal impetus of reproductive and regenerative medicine and the rapid translation of experimental procedures into clinical practice. In analogy to SCNT, chromosomes from a patient's egg cell can be introduced into an enucleated healthy donor egg cell in order to avoid transmission of defects connected to mutations of mitochondrial DNA.^8 ^Recently, the UK Human Fertilisation and Embryology Authority published a report on the technique, referred to as 'three-parent IVF' because the offspring would have a small amount of the egg donor's genetic material and therefore three genetic parents [22]. Although further research is needed, researchers are already pressing the government to prepare legislation that would make the procedure legal for translation into clinical practice [23].

## Israel and Austria

### ART utilization

In both Israel and Austria the first IVF birth was reported in 1982, and subsequently ART services have become the standard of care. 24 IVF centers have been operating since 2002 in Israel [24], whereas in Austria, there were 25 operating clinics in 2006 [25], and 27 in 2009 [26]. There is no available official data specifically on egg cell donations or implantations. However, differences in utilization rates and the extent of public funding for ART in general can be seen from the following data.

Israel is known for its high rates of ART utilization and innovative clinical practices. It boasts by far the highest rates of intervention in the world, measured by the number of IVF treatment cycles per capita [27,28]. In Austria, reproductive interventions are far less acceptable, and ART utilization rates are lower:

• In 2002, the total number of IVF cycles in Austria was 4,680 (including ICSI and frozen embryo transfer)[29] compared with 20,886 in Israel [24].

• In Austria, for 2004, 658 live births following ART were reported [27], whereas for the same year in Israel, Ministry of Health (MoH) data indicated 3,574 live births^9 ^[24].

• In Austria, ART live births accounted for about 1.3% of all births in 2008 [26], compared with 3% in Israel [24].

Austria and Israel also differ in the scope of public funding. In Austria, in 2008, 289 of the 1,039 ART live births were in public clinics while 750, the majority, were in private clinics [26]. Under Austria's IVF Fund Act, 1999 (*IVF-Fonds-Gesetz*) subsidies cover 70% of treatment costs for a maximum of four cycles in eligible clinics (both public and private), with a possibility of additional cycles of treatment if pregnancy was achieved within the first four [30]. In Israel, funding under the National Health Insurance (NHI) Law, 1995 covers unlimited IVF treatment cycles for up to two children within the current relationship.

Austrian law does not allow egg cell donation, as we shall see, but in Israel, clinical guidelines for public funding of IVF, issued in 1998, recommended that women undergoing IVF with their own eggs would be eligible for public funding only up to the age of 45 years, while the age limit for women undergoing IVF with donor eggs was set at 51 [31]. The Eggs Donation Law, 2010 extended the age of eligibility for accessing treatment by another three years, to 54. It is yet to be seen whether public funding guidelines will be adjusted, or whether the gap between the Law and the guidelines will create a new market for private medical practice.

There are multiple cultural factors that explain the differences in the use and public funding of ART in Israel and Austria. Israeli doctors are leaders in ART research and development. Jewish tradition places high value on the religious commandment to be fruitful and multiply, and the family is a central institution in social life. Israeli individuals and society have a general propensity to accept and consume technological novelties. Demographic policy is pro-natal against the historic backdrop of the Holocaust and in the context of the Israeli-Palestinian conflict [32-34]. And the Supreme Court has struck down restrictions on access to ART under Israel's IVF regulations [35] on more than one occasion, on grounds of a constitutional right to parenthood [36,37].

On the other hand, Austria has a Catholic tradition that views the fertilised egg as a human being deserving protection. Like in other western European countries, fertility rates are below replacement level and women have the freedom to choose not to have children. In general, Austrian society is cautious about scientific progress and due to the history of the Nazi eugenics, the political culture is sensitive to technical interventions at the beginning of life. According to a 2010 survey, it is the least optimistic among European countries about biotechnology [38]. In addition, Austria's medical system is still predominantly public, and the influence of private biomedicine and the biotechnological industry is small compared to other western countries. These factors combine to explain that there is less interest in and demand for ART in Austria compared with Israel.

At the same time, in both Israel and Austria, there has been a steady increase in ART utilization rates over the past decade (Table 1). In Israel, the total number of treatment cycles rose by 56%, from 2001 to 2009, together with an increase in the per capita ratio^10 ^by 38% [24]. Over the same period of time, in Austria, there were similar increases. Treatment cycles rose by 39%, and the per capita ratio increased by 36% [26]. These data correspond to observations from other countries in Europe [25].

**Table 1:** Increase in ART utilization over the past decade

	Israel		Austria	
**year**	**no. of cycles**	**per capita ratio**	**no. of cycles**	**per capita ratio**
2001	20,512	12.9	4,726	2.37
2009	31,978	17.8	6,599	3.22

Data for Israel are found in ref. 24; data for Austria were taken from ref. 26. The per capita ratio is the number of treatment cycles per 1,000 women between the ages of 15 and 49. Counts of Austrian women of the respective age group are found on www.statistik.at.

### Regulation of egg cell donation

#### (a) Israel

Prior to the enactment of Israel's Eggs Donation Law, 2010 [39], the IVF Regulations allowed egg cell donations only by women who were undergoing IVF as infertility treatment. The rationale was that the health risks could not be justified unless the intervention was undergone primarily for the donor's own benefit. But given the difficulty in obtaining human egg cells, infertility patients ordinarily prefer to fertilize and preserve for their own use all the eggs retrieved in a given cycle. The discrepancy between the reluctance of patients to donate eggs and the increasing demand for donations led to a so-called 'shortage'. Private clinics started offering economic inducements to infertility patients to donate eggs, by waiving certain costs of treatment if they would agree to 'share' their eggs with others [40]. However, this source dried up for all practical purposes after the exposure, in 2000, of a scandal that came to be known as the 'eggs affair'.

The affair came to light when some women filed a personal injury action claiming damages from one of Israel's leading fertility experts, who was a chief of gynecology at one of its largest public hospitals. The plaintiffs alleged that the doctor submitted them to excessive hormonal stimulation, retrieved dozens of eggs from single treatment cycles, and used these eggs in the treatment of large numbers of recipients at a private clinic - without their informed consent. The case was eventually settled out of court, but the plaintiffs had also complained to the police, and this led to a criminal investigation [41]. The criminal proceedings culminated in a plea bargain, according to which the doctor confessed to certain facts and the matter was referred to a professional disciplinary tribunal, which eventually suspended his medical license for a period of two and a half years. Because the case was not adjudicated in a court of law, and the decisions of the disciplinary court are not made public, the full facts of the affair were never made clear. According to journalist reports, the doctor confessed that in one case he removed 232 ova from one patient and used 155 of them for 33 recipients, and in another, he took 256 ova and used 181 for treating 34 other women [42].

A crisis of trust between infertility patients and their doctors resulted, and the practice of eggs donation in Israel ceased almost entirely. At the time, it was estimated that 2,000 women in Israel were waiting for a donation [43]. The MoH responded by appointing an ad hoc committee which concluded its deliberations in 2001 and recommended legislation to allow donations by healthy volunteers [44], but it took almost ten more years until the Eggs Donation Law was enacted in 2010. Meanwhile, the practical solution to the local shortage was to allow (or create) a cross-border flow of human egg cells from other countries in which there were no legal restrictions on young healthy woman acting as donors, or on paying them to do so. Thus, the MoH amended the IVF Regulations so as to permit the use of egg cells imported from other countries [45]. Nonetheless the numbers of women in need continued to grow. By 2007, when the legislature took up the governmental initiative to enact a law that would allow donation by healthy volunteers inside Israel, the numbers of women waiting for egg donations was estimated at 6,000 by a patient organization advocate [46], a three-fold increase since the start of the public debate in 2000.

In addition there was a new demand of scientists for egg cells for the purpose of cloned stem cell research. While Israel's anti-cloning statute prohibits reproductive cloning [47], stem cell research with embryos cloned from human egg cells is not forbidden, subject to approval by an ethics review committee. However, under the IVF Regulations, egg cells taken from a woman's body could be used only for the purpose of reproduction, and this precluded their use for research. Consequently, in 2003, the committee vested with advisory authority under the anti-cloning statute called for a change in legal regulation that would allow the donation of egg cells for research [48]. In view of this, the MoH put on hold the draft legislation proposed in 2001, to allow egg cell donations by healthy volunteers for infertility treatment, so as to prepare a comprehensive legislative proposal that would include donations for research as well. The bill was published in 2007 [49] and eventually enacted by the Knesset in 2010.

The essence of the Eggs Donation Law, 2010 is to allow donations from healthy volunteers, primarily for infertility treatment, but the donor may designate up to 2 egg cells (or 20% of the total number of those retrieved in any given cycle) either for research or to be frozen for her own future use. In this respect the Law is permissive, but at the same time it subjects the practice of egg cell donation to detailed regulation. The donor must be between 21 and 35 years old, and she may undergo no more than three retrieval cycles, spaced at intervals of at least 180 days. There can be no more than two recipients from each retrieval procedure, so that all in all no more than six children can be born from any one donor. As mentioned above, there is an upper limit on the age of the recipient - no more than 54 years of age. The Law clarifies that the offspring is the legal child of the recipient and that the donor has no parental rights or responsibilities. While trading in egg cells is prohibited (section 8), the state will pay donors 'compensation' in an amount that is to be determined by the Minister of Health (section 43).

Furthermore, the Law imposes restrictions on the freedom of individual donors and recipients and subjects them to various technocratic mechanisms that are intrusive of privacy. For example, a donor may not be a married woman; she may not be related to the recipient; and the donation will be anonymous (section 13), unless special permission is obtained from an 'exceptions committee' (section 22). Both the recipient and the donor must submit a formal request - to a 'responsible physician' or the 'approvals committee', respectively (sections 11,12). The Law also establishes a 'data base' to keep track of the number of donations from each woman and to rule out any biological relation between the donor and the recipient (section 30), as well as an 'infants registry', mainly in order to preclude half-sibling marriage (section 33);^11 ^but neither the adults involved in the reproductive collaboration nor the children born as a result have a right to receive any identifying information from these sources.

#### (b) Austria

The law in Austria on MAR has been characterised as 'restrictive if not hostile' [50]. The Act on Reproductive Medicine (*Fortpflanzungsmedizingesetz*), 1992 [51] allows the use of ART only within marriage or a stable heterosexual civil partnership^12^, and prohibits egg cell donation. Sperm donation can be used only by couples where the male partner is infertile, but not in combination with IVF, and is not available at all to single women or lesbian couples. In other words, IVF in Austria may be used only by a married or cohabiting heterosexual couple with their own gametes. Women must be no older than 40, and men - 50, when starting the treatment. The Act also provides that the mother of any offspring from IVF is the woman who carried the pregnancy, which excludes surrogate motherhood arrangements. Recent discussion of egg cell donation by Austria's Bioethics Commission revealed controversy over issues related to the commodification of egg cells and the exploitation of donors [52].

Austria is also regarded as one of the countries with the most restrictive laws on embryo research in Europe [53]. The Act on Reproductive Medicine states that human embryo cells capable of development may be used only for reproduction, and prohibits the creation of human embryos for research purposes as well as the procurement of stem cells from 'surplus' IVF embryos (i.e., embryos that will no longer be used by their parents and would otherwise be discarded) [54]. Therefore, research on embryonic stem cells is not well established in Austria. At the same time, the Act does not expressly prohibit the import and use of embryonic cell lines, Austrian scientists are participating in EU funded projects of embryonic stem cell research [52,55], and funding through the Austrian Science Fund is not restricted for this research field as a matter of principle. There is currently a discussion as to whether 'surplus' embryos from IVF can be used for research with the informed consent of the parents. Embryos created by means of SCNT or parthenogenesis could also be used to establish stem cell lines, at least in theory, as these embryos would not have the potential to develop into a child [52]. Nonetheless, there is no demand in Austria for egg cells for research, and their use for purposes other than fertilization raises ethical reservations.

In 1998 two married couples living in Austria and suffering from infertility challenged the constitutionality of the Act on Reproductive Medicine in a petition to its Constitutional Court, arguing that the prohibition of IVF with donated sperm or egg cells infringed the basic rights to privacy and to found a family guaranteed by the European Convention on Human Rights [56]. For the purposes of this paper we focus on egg cells. In 1999, the Court gave its decision, finding that the Act did interfere with the applicants' rights, but that the interference was justified in view of the moral and ethical implications and the best interests of the child-to-be [50]. Subsequently, in 2000, the couples applied to the European Court of Human Rights, which delivered its decision, in SH v Austria, in 2010 [57].

Austria argued that even though the right to respect for private life encompasses the right to fulfil the wish for a child, it does not follow that the State is under an obligation to permit indiscriminately all technically feasible means of reproduction. Austria's legislation was designed to avoid the forming of 'unusual' personal relations such as a child having more than one biological mother (a genetic one and one carrying the child). A third party submission by Germany argued that 'split motherhood' was an absolute novelty in nature and the history of mankind, and it posed a serious threat to the welfare of the child. German law also prohibits egg cell donation [58], and the intention is to protect the child's welfare by ensuring the unambiguous identity of the mother. The child would have difficulty coping with the fact that two women had a part in his or her biological existence. This ambiguity might jeopardize the development of the child's personality and self-identity. Another danger was that of conflict between the two mothers, to the detriment of the child.

Austria argued further that the aim of its law was to prevent 'exploitation and humiliation of women, especially those from economically disadvantaged backgrounds', who might be pressured to donate egg cells to other infertile women so as to receive IVF treatment which they could not afford otherwise.^13 ^It was also concerned about the risks of commercialization and the use of gamete donation for the 'selection' of children. Additionally, children had a legitimate interest in information about their descent, while with donated egg cells, the actual parentage of a child would not be revealed in the register of births.

The European Court noted that the state parties to the European Convention on Human Rights enjoyed a wide margin to regulate ART in view of the sensitive moral issues raised with the fast-moving medical developments related to IVF. Within this margin of discretion a state may prohibit ART altogether, but once it permits some of the technological applications it must conform with the principle of equality. Moral considerations or concerns about social acceptability were not sufficient justification to ban a specific technique such as egg cell donation. The risks, including that of the exploitation of women, could be minimized by less restrictive safeguards, such as the prohibition of remuneration for donation. The Court acknowledged that the certainty of motherhood - *mater semper certa est *- was a basic principle of civil law, but noted that family relations which do not follow the typical biological parent-child relationship, such as adoption, are not new. As for the legitimate interest of individuals to know their actual parentage, this was not an absolute right and could be balanced with the competing interest of donors in anonymity. The Court concluded, by a majority opinion, that it could not justify the difference in treatment between couples who needed egg cell donation and those who did not.^14^

## Cross-border egg cell donations

Despite the differences between Israel and Austria with regard to the law, policy and practice of ART, limited local access to egg cells in both countries has led to the emergence of cross-border markets. Israel has been actively involved in 'reproductive tourism' related to egg cell donations. In Austria, the issue is less visible, but women there also use cross-border services, and private fertility clinics offer egg cell donation to their clients in cooperation with partner clinics in other European countries, e.g. Spain and the Czech Republic. These practices are part of a flourishing global market for egg cells, where transnational IVF clinics broker sales between generally poor, female vendors and wealthy purchasers, beyond the borders of national regulation and with little clinical or bioethical scrutiny [7].

Like other medical tourism practices that lie on a spectrum between life style spas and cosmetic services, trafficking in organs for transplantation and the fraudulent marketing of unproven stem cell treatments [59], transnational egg cell donations - and surrogacy too - are driven by differences in law and policy between countries and economic gaps [55,60]. For example, 'pregnancy contracts' are being outsourced to India where private fertility clinics are offering surrogate mother services as part of a $ 2.3 billion medical tourism 'industry' because of the low costs and lack of protective regulation [61,62]. IVF procedures in the unregulated Indian clinics generally cost a fraction of what they would in Europe or the U.S., with surrogacy as little as one-tenth the price [63].

A recent study of six European countries estimated that between 11,000 to 14,000 patients per year were seeking cross-border ART services for between 24,000-30,000 treatment cycles, because of legal restrictions in the country of origin. For example, single and lesbian women from France, Norway and Sweden go abroad for sperm donation, because it is not legally accessible to them in their home countries. Women from Germany seek egg cell donations abroad because they are illegal in their home country. Women from England also travel abroad for egg cell donations - because of waiting lists [55]. Differences in economies and payments to egg cell donors also drive this reproductive tourism. In the USA payment to donors in the sum of 5,000 USD is standard [64], while sums of 900 Euro in Spain or 500 Euro in the Czech Republic have been reported [65], and women at an Israeli clinic in Romania earned as little as 200 USD per retrieval cycle [66].

The record of Israel's involvement in extra-territorial egg cell donation practices is illustrative of the modus operandi of the global market. As mentioned above, following the cessation of donations by infertility patients in the wake of the 'eggs affair', the IVF Regulations were amended so as to permit the use of imported egg cells. The amendment allowed doctors "to implant an egg retrieved and fertilized outside Israel, in the body of a woman in Israel" [45]. This meant that the egg cells would be provided by women at a facility outside Israel: the sperm of the male partner could be frozen in Israel and transported to the facility abroad; there the donated egg cells would be fertilized with the sperm; and the fertilized egg could then be frozen and transported back to Israel for implantation in the female partner. The MoH also authorized four clinics in Israel to engage in these procedures after examining and approving the clinical conditions and laboratory methods of their collaborating clinics in Romania and the Ukraine [67].

However, success rates of treatment with frozen embryos are lower than with freshly fertilized eggs. Much as medical tourism for kidney donations increased when it became known that transplantation success rates were higher with organs from live donors rather than from cadavers, so too the evidence-based benefits of egg cell donation technology gave rise to medical entrepreneurism in the cross-border market. Israeli doctors started advising their female patients to travel abroad to collaborating clinics where they would be implanted with freshly fertilized donated eggs. Sometimes the same doctors set up the IVF facility abroad as a private enterprise that would service their Israeli patients as well as those of the local population who could afford the fees. Often doctors would accompany their patients and perform the treatment at the clinic abroad. The website of one prominent private IVF center explained the package deal: a woman who wished to have an egg donation would need to travel abroad for implantation of the embryos, to the clinic where the egg cells were donated and fertilized; the flight there and back would be together with other patients and accompanied by a doctor from the center who would also perform the implantation. "All air travel arrangements, plane tickets, taxes, transfers and full pension hotel accommodation are taken care of by center staff and included in the cost of treatment" [68]. The cost to patients was in the range of several thousand Euro or USD [66,69].

When Israel allowed the use of egg cells from abroad because its own women were refusing to donate, it applied a double standard and turned a blind eye towards dubious practices that occur outside its territory in less regulated countries. Moreover, the blurring of medicine as professional practice and business creates a conflict of interests: between doctors' fiduciary relationship with the patient and their ethical duties of beneficence and non-maleficence, on the one hand, and their economic interest in profiting from the business, on the other. The ethical compromise is expressed in the testimony of a spokeswoman for an egg donation web forum during the parliamentary debate around the Eggs Donation Law. She testified that she was going for an egg donation in Eastern Europe for the tenth time. "It's not pleasant to say so", she said, "but there is good livelihood for lots of good and respectable doctors." ([43], per Anonymous)

Clinics in public hospitals found that in order to keep their patients, they had to compete with these private medical practices, and also set up IVF facilities, mainly in Eastern Europe. Indeed, in July 2009, yet another 'eggs affair' shook Israel, this time with a police raid of an IVF clinic in Romania, and the detention for questioning of doctors, patients and management staff under suspicion of involvement in human egg and stem cells trafficking. Two of the doctors were employees of a government-owned hospital in Israel, and bedding from that hospital was being used in the Romanian clinic. Three years earlier, a MoH official had written a letter of warning that the clinic was no longer operating with a license from the Romanian authorities [70-72].

These patterns are typical of reproductive tourism in general and not unique to Israel, but Israel stands out in its designation of public funds to support these practices. In 2005, the MoH issued a circular to clarify that the health funds were obliged to provide egg cell donation services outside Israel within the coverage of the NHI [73]. It was not entirely clear whether the administrative directive applied only to cases in which the fertilized egg was imported for implantation in Israel, in accordance with the IVF Regulations, or whether it also obligated the health funds to cover the costs when the woman travelled for implantation abroad. In any event, health funds may provide universal services above and beyond their legal obligations under the NHI Law, and it seems that prior to the Romanian scandal they participated in the costs of donation abroad whatever the circumstances. In 2008, according to a patient rights organization website, "because there are no egg donations in Israel" all the health funds participated in the costs to the sum of approximately $2,000 [74]. At least one of the health fund supplementary insurance programs covered most of the expenditure for up to two egg donations outside Israel, including when the implantation was performed there [75]. Israel's generosity in funding infertility treatments has been criticized as excessive, because it encourages women to undergo unlimited cycles of IVF treatment with the attendant health risks, and because of questions as to funding priorities in relation to other pressing public health needs. When cross-border IVF is practiced, however, the question of public funding gains an additional moral dimension.

## Conclusion

Egg cells are a precious human tissue, due to their scarcity, biological complexity and economic value. They can be procured only by means of sophisticated ART that is intrusive, invasive and risky for the women who are involved. Therefore the use of egg cells for both infertility treatment and stem cell research is wrought with ethical conundrums. This paper has described the dramatic developments that unfolded around the subject in Israel and Austria. The two countries represent extremes on the spectrum of the ethical debate around the acceptability of egg cell donations. They differ in utilization rates, public funding policies and legal regulation of ART, but in both there has been a steady increase in domestic usage and the emergence of cross-border markets developed by medical entrepreneurs, driven by global economic gaps, made possible by trans-national regulatory lacunae and finding expression as consumer demand.

The need of post-menopausal women for egg donations appears to be a major factor in the growth of cross-border markets in latter years. The market mentality is not really appropriate for the collaborations that are necessary in order to bring a child into the world. Yet some medical entrepreneurs involved in the business of egg cells appear to view women's bodies as a natural resource to be mined for profit. It is not surprising that egg cells are not readily available, and there is a so-called 'shortage'. It is an artificial shortage created by the technological possibility and the demand of 'consumers'. This demand arises within a complex cultural context, in which the technological possibilities generate new social constructs of infertility and hence, new forms of suffering from childlessness. On the one hand, women are appropriating the technology so as to experience pregnancy and childbirth even though they cannot be genetic mothers. On the other hand, the satisfaction of their desire comes at substantial cost. The transnational market in egg cells for infertility treatment and research raises a multitude of ethical issues related to the commercialization of medicine, the commodification of human body parts, and the exploitation of women. Other issues of paramount concern relate to the rights of the children, such as the right to know their genetic origins, and the right to naturalization and to enter the country of the commissioning parent's origin.

The proliferation of ART, in general, and its use with egg cell donations, in particular, has been driven by a rapid translation of medical experimentation into marketed services. In other words, egg cells were 'harvested' on a wide scale before the science and technology were optimized. No doubt there have been benefits. Nonetheless, concerns about the long term effects on the health of children and women in various contexts of IVF are only now coming to light. There is by now ample evidence that these risks are considerable and that there is a problem of under-reporting adverse effects. For example, the possible immune reaction of a recipient towards embryos from donated egg cells has been completely ignored, but recent studies show that serious hypertensive disorders (pre-eclampsia) - a classical case of mal-adaptation of the mother's immune system to the fetus - occur with increased incidence in IVF using donated egg cells [76,77].

Since much of the practice of egg cell donations takes place in the private market, there is a general lack of medical and epidemiological data on its efficacy and safety. If that lack is to be filled, there is need for harmonisation of domestic laws and formulation of new instruments for international governance that will require transparency and accountability from professionals. Israel and Austria have very different approaches of law and policy to egg cell donation but cross-border issues are common to both, and it is clear that the global ventures of medical entrepreneurs pose new challenges for transnational governance. This paper indicates the scope of the matter and the need for a broad and concerted global response. An argument could be made for extra-territorial criminal jurisdiction. At the very least, a state ought not to support cross-border IVF practices with public funds unless it assumes responsibility to take appropriate measures that will ensure they do not lead to abuse.

## Abbreviations

ART: Assisted reproductive technology; IVF: *In vitro *fertilisation; MAR: Medically assisted reproduction; MoH: Ministry of Health; NHI: National health insurance; SCNT:Somatic cell nuclear transfer.

## Competing interests

The authors declare that they have no competing interests.

## Authors' contributions

Both authors have made substantial intellectual contributions to this article in the conception, design and interpretation of the data, the drafting and the final approval of the submitted version.

## Authors' information

CS is a human rights bio-ethicist. She works as an independent consultant on health and research ethics for local and international public interest organisations, and teaches at the law faculty of Haifa University.

GWF is a professor of Medical Biochemistry working at the intersection between basic biomedical research and bioethics and the founder of Ethucation - the Austrian unit of NIMED, the Network of Institutions for Medical Ethics Education, UNESCO Chair in Bioethics (IL).

## Endnotes

^1 ^ICSI entails the isolation of one sperm cell from the semen and injecting it directly into the woman's ova.

^2 ^In this paper we use the term 'egg cell' to replace the interchangeable terms of 'egg', 'oocyte' or 'ovum'.

^3 ^The numbers are based on the records of the statutory approvals committee under the Surrogate Mother Agreements (Approval of the Agreement and Status of the Child) Law, 1996 since the law came into force, and were presented by Etti Samama, a researcher from Ben Gurion University, at a conference held in Tel Aviv on December 27, 2010.

^4 ^Note that follicular development, which is a necessary support mechanism for egg cell maturation, is itself a complex and not fully understood biological process.

^5 ^Somatic cell nuclear transfer (SCNT) is a cloning technique for producing a genetically identical duplicate of an organism by replacing the nucleus of an unfertilized egg cell with the nucleus of an adult cell and then stimulating it to develop. Cloning for reproductive purposes is forbidden under Israeli law and in many other countries. So-called therapeutic cloning - i.e., stem cell research with cloned embryos for potential use in transplantation medicine - would eliminate problems related to host immune rejection of the grafted tissue.

^6 ^Pluripotency refers to the potential of a stem cell to differentiate into any type of fetal or adult cell. Induced pluripotent stem cells, commonly abbreviated as iPSCs, are adult cells that have been reprogrammed so that they revert to an embryonic-like state and regain differentiability. Tissues derived from iPSCs would be a nearly identical match to the cell donor and thus probably avoid rejection by the immune system, like cloned stem cells. Hence the hope of researchers that they will prove useful in personalised regenerative medicine provided that general drawbacks of pluripotent cells, like tumorigenicity, can be overcome.

^7 ^Parthenogenesis is asexual production, where the development of an embryo from an egg cell occurs without fertilization by a male. Blastocyst refers to the stage of development when the egg cell has become a cluster of embryonic cells that is ready to implant in the lining of the womb. Parthenogenic blasotcysts can be generated from mature egg cells by stimulation in vitro. In humans they will not develop into viable embryos.

^8 ^Each cell contains a general genome in its nucleus (nuclear DNA) and an extra genome in its mitochondria, the vesicles of cells responsible for energy generation through respiration. A variety of diseases is connected to mutations in mitochondrial DNA and could thus be avoided.

^9 ^The number is calculated as follows: 23,828 cycles × 15% live births per cycle = 3,574 live births.

^10 ^Treatment cycles per 1,000 women between the ages of 15 and 49.

^11 ^Concerns about unwitting half-sibling marriage are universal. In Israel these concerns are raised primarily by rabbinical authorities because the law of marriage is predominantly governed by *halakha *for the Jewish majority in the country. There is controversy amongst *halakhic *authorities as to the marital eligibility of children born of third-party reproduction, due to doubts about *mamzerut*. This lies beyond the scope of this paper.

^12 ^Civil partnership is possible in Austria since 2010.

^13 ^The assumption is that the donor will herself be in need of treatment, as was the case in Israel before the enactment of its new law.

^14 ^Austria's appeal against this ruling is pending before the European Court's Grand Chamber. On February 23, 2011, the Chamber decided to examine the merits of the claim of violation of privacy, but rejected the argument that the right to found a family guarantees a right to procreation. [http://cmiskp.echr.coe.int/tkp197/view.asp?action=html&documentId = 826894&portal=hbkm&source=externalbydocnumber&table=F69A27FD8FB86142BF01C1166DEA398649]

## Supplementary Material

Additional file 1 **Health risks of egg cells procurement**. Click here for file
