# Supplementary material for: Patterns of globalized reproduction: Egg cells regulation in Israel and Austria
Source: Isr J Health Policy Res. 2012 Apr 18;1:15. doi: 10.1186/2045-4015-1-15 (PMC3424961; doi:10.1186/2045-4015-1-15)
Supplement: Additional file 1 — Health risks of egg cells procurement. [file 2045-4015-1-15-S1.DOC]

**Additional file 1**

Israel Ministry of Health and Israeli Medical Association, 1996.

**IVF Consent Form**[[1]](#footnote-2)

**Health risks of egg cells procurement**

**The Risks of Hormone Therapy:**

Hormone therapy often leads to hyperstimulation of the ovaries. Such hyperstimulation is usually mild and manifests in swelling of the abdomen, abdominal pain, enlargement of the ovaries and even slight accumulation of fluid in the abdomen. These symptoms and signs subside approximately 3 – 4 weeks after the eggs are removed, but if conception is achieved they may last longer. Rest and increased intake of fluids usually suffice as treatment, and hospitalization is usually not required.

Moderate or severe hyperstimulation are very rare. Moderate hyperstimulation includes, in addition to the aforementioned, nausea, diarrhea and over-concentration of the blood. Severe hyperstimulation (0.5% - 5%) also involves the risk of pulmonary infiltrations and emboli. Additional rare complications include heart and/or kidney failure. Isolated cases of limb amputations and even death have been reported.

It is important to note additional rare complications, such as ovarian torsion, rupture or hemorrhage. These complications necessitate surgical intervention (open or laparoscopic) to release the torsion. In rare cases, the need to resect [i.e., remove – CS] the ovaries has been reported.

Solitary reports have recently appeared in the medical literature, examining the possibility of an increase in the incidence of ovarian cancer following treatment with ovulation stimulating agents. … [I]nformation is still accumulating and the conclusions will only be available in several years.

When treatment includes premature suppression of the ovaries, side effects similar to those of menopause may occur. In addition, ovarian cysts may develop, requiring their removal and/or discontinuation of treatment. Allergic reactions to the hormone medications are rare.

…

**The Risks of Egg Retrieval:**

The egg retrieval procedure entails discomfort and even pain, and following the procedure a few hours of rest are required. The main risks associated with the insertion of a needle into the ovary are infection and hemorrhage. Pelvic infection is rare and usually resolves with antibiotic treatment.

At times, surgery is required to drain an abscess or resect [i.e., remove – CS] damaged fallopian tubes or ovaries. Infections reduce the chances of conceiving. In rare cases, complications of the infection may necessitate resection of the uterus. Mild hemorrhage occurs during most manipulations of the ovary. Infrequently, the hemorrhage is more extensive and requires blood transfusions, procedures to stop bleeding, and resection [i.e., removal – CS] of the ovaries and uterus. Damage to the intestine is rare but possible.

…

**The Risks of Anesthesia:**

Anesthesia is a relatively safe procedure, but it does involve certain risks.

The risks of local anesthesia are various degrees of allergic reactions to the anesthetic drugs. The risks of epidural anesthesia are allergic reactions and damage to the nerves of the lower body. The risks of general anesthesia include damage to the teeth and/or vocal cords due to the introduction of a tube into the windpipe, various degrees of allergic reactions to the anesthetic drugs and in very rare cases, death. Therefore, it is essential to report any known sensitivity to medications in advance. With general anesthesia there is also a risk of aspirating gastric content. Fasting for 6 hours at least reduces the risk of aspiration.

1. <http://www.ima.org.il/FilesUpload/DocumentInformedConsent/0/1/788.doc> (accessed 30 January 2011) [↑](#footnote-ref-2)
